# [4-(1-Benzofuran-2-yl)phen­yl]diphenyl­amine

**DOI:** 10.1107/S1600536810050439

**Published:** 2010-12-11

**Authors:** Ping-Hsin Huang, Kai-Ling Lin, Yuh-Sheng Wen

**Affiliations:** a Cardinal Tien College of Healthcare & Management, Taipei, Taiwan 231; b Institute of Chemistry, Academia Sinica, Nankang, Taipei, Taiwan

## Abstract

The asymmetric unit of the title compound, C_26_H_19_NO, contains two mol­ecules. The dihedral angles between the benzofuran and benzene rings are 5.09 (8), 59.02 (8) and 67.74 (8)° in one mol­ecule and 18.70 (8), 52.78 (8) and 41.74 (8)° in the other. Weak inter­molecular C—H⋯π inter­actions help to stabilize the molecular structure .

## Related literature

The title compound is a precursor for the production of hole transporting and/or emitting materials, see: Shen *et al.* (2005 ▶). For lone-pair delocalization, see: Wang *et al.* (2001 ▶). For a related structure, see: Bak *et al.* (1961 ▶).
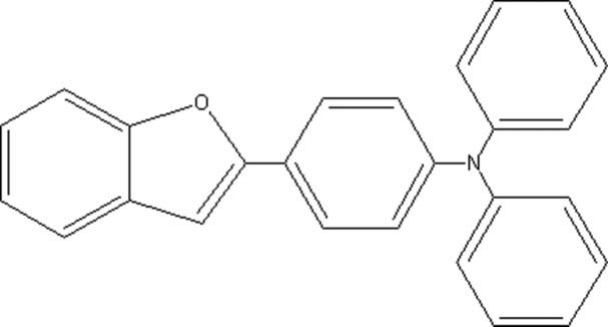


## Experimental

### 

#### Crystal data

C_26_H_19_NO
*M* *_r_* = 361.42
Triclinic, 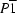

*a* = 10.1804 (6) Å
*b* = 12.0198 (7) Å
*c* = 16.0191 (10) Å
α = 91.752 (3)°
β = 101.606 (3)°
γ = 104.400 (3)°
*V* = 1853.07 (19) Å^3^
*Z* = 4
Mo *K*α radiation
μ = 0.08 mm^−1^
*T* = 100 K
0.38 × 0.32 × 0.18 mm

#### Data collection

Bruker SMART CCD area-detector diffractometer
13657 measured reflections
6509 independent reflections
4745 reflections with *I* > 2σ(*I*)
*R*_int_ = 0.028

#### Refinement

*R*[*F* ^2^ > 2σ(*F* ^2^)] = 0.045
*wR*(*F* ^2^) = 0.115
*S* = 1.08
6509 reflections
506 parameters
H-atom parameters not refined
Δρ_max_ = 0.57 e Å^−3^
Δρ_min_ = −0.27 e Å^−3^

### 

Data collection: *SMART* (Bruker, 2001 ▶); cell refinement: *SAINT* (Bruker, 2001 ▶); data reduction: *SAINT*; program(s) used to solve structure: *SHELXS97* (Sheldrick, 2008 ▶); program(s) used to refine structure: *SHELXL97* (Sheldrick, 2008 ▶); molecular graphics: *ORTEP-3 for Windows* (Farrugia, 1997 ▶); software used to prepare material for publication: *WinGX* (Farrugia, 1999 ▶).

## Supplementary Material

Crystal structure: contains datablocks global, I. DOI: 10.1107/S1600536810050439/vm2056sup1.cif

Structure factors: contains datablocks I. DOI: 10.1107/S1600536810050439/vm2056Isup2.hkl

Additional supplementary materials:  crystallographic information; 3D view; checkCIF report

## Figures and Tables

**Table 1:** Hydrogen-bond geometry (Å, °) *Cg* is the centroid of the C61–C66 ring.

*D*—H⋯*A*	*D*—H	H⋯*A*	*D*⋯*A*	*D*—H⋯*A*
C15—H15⋯*Cg*	0.93	2.97	3.787 (2)	147

**Fig. 1.**
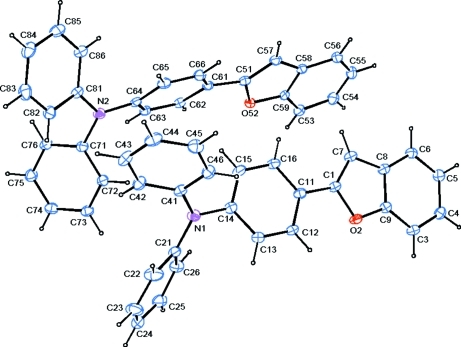
Molecular structure of (I) with 30% probability displacement ellipsoids, showing the atom-numbering scheme employed. H atoms are shown as small spheres of the arbitrary radii.

## supplementary crystallographic information

### Comment

The title compound, (I), has been shown to be an precursor for the production of hole transporting and/or emitting materials (Shen *et al.*, 2005). One pot synthesis of a benzofuran derivative with a 2-substituent has been achieved by the Pd complex catalyzed Sonogashira coupling reaction of 2-iodophenol with terminal alkynes, followed by cyclization of the internal alkynes formed. The molecular structure is shown in Fig. 1. The dihedral angle between the benzofuran and benzene rings is 5.09 (8)° (C11—C16), 59.02 (8)° (C21—C26), and 67.74 (8)° (C41—C46) [18.70 (8)° (C61—C66), 52.78 (8)° (C71—C76), and 41.74 (8)° (C81—C86) for the second molecule]. There are no significant C—H···O hydrogen bonding interactions between molecules. Weak intermolecular C—H···π interactions help to stabilize the crystal structure. As shown in Fig. 1, the three phenyl rings of the amino group are arranged in a propeller-like, non-coplanar fashion. The pyramidalization of the NC_3_ core is weak, the N lone pair may be delocalized, mainly toward the benzofuran and increase the conjugated strength (Wang *et al.*, 2001).

### Experimental

The compound was synthesized by the following procedure. A two-necked round-bottomed flask was charged with PdCl_2_(PPh_3_)_2_ (100 mg), (4-ethynyl-phenyl)-diphenyl-amine (1.55 g, 5.46 mmol), CuI (30 mg), 2-iodophenol (1.00 g, 4.55 mmol), triethylamine (1.3 ml), and DMF (10 ml), and the reaction mixture stirred under nitrogen and heated at 333 K for 24 h. After cooling, the mixture was diluted with diethyl ether and the organic phase was washed with water and brine. After drying over anhydrous MgSO_4_ and removing the volatiles, the residue was purified by column chromatography using CH_2_Cl_2_/n-hexane as eluent, followed by recrystallization from CH_2_Cl_2_ and hexane to yield 0.60 g (37%) of (I) as a white solid. Crystals suitable for X-ray diffraction were grown from a CH_2_Cl_2_ solution layered with hexane at room temperature. ^1^H NMR (CDCl_3_): 7.69 (d, 2 H, J = 8.47 Hz), 7.54 (d, 2 H, J = 7.84 Hz), 7.32–7.24 (m, 7 H), 7.08 (tt, 7 H, J = 8.65 Hz), 6.88 (s, 1 H). FAB MS (m/e): 361.1 (*M*^+^) Anal. Calcd for C_26_H_19_NO: C, 86.40; H, 5.30; N, 3.88. Found: C, 86.54; H, 5.38; N, 3.78.

### Refinement

H atoms were located geometrically and treated as riding atoms, with C—H = 0.93 Å, and with *U*_iso_(H) = 1.2*U*_eq_(C).

### Crystal data

**Table:**

C_26_H_19_NO	*Z* = 4
*M**_r_* = 361.42	*F*(000) = 760
Triclinic, *P*1	*D*_x_ = 1.295 Mg m^−^^3^*D*_m_ = 1.295 Mg m^−^^3^*D*_m_ measured by not measured
Hall symbol: -P 1	Mo *K*α radiation, λ = 0.71073 Å
*a* = 10.1804 (6) Å	Cell parameters from 2733 reflections
*b* = 12.0198 (7) Å	θ = 2.3–24.1°
*c* = 16.0191 (10) Å	µ = 0.08 mm^−^^1^
α = 91.752 (3)°	*T* = 100 K
β = 101.606 (3)°	Prism, colourless
γ = 104.400 (3)°	0.38 × 0.32 × 0.18 mm
*V* = 1853.07 (19) Å^3^	

### Data collection

**Table:**

Bruker SMART CCD area-detector diffractometer	4745 reflections with *I* > 2σ(*I*)
Radiation source: fine-focus sealed tube	*R*_int_ = 0.028
graphite	θ_max_ = 25.0°, θ_min_ = 1.3°
phi and ω scans	*h* = −12→11
13657 measured reflections	*k* = −14→14
6509 independent reflections	*l* = 0→19

### Refinement

**Table:**

Refinement on *F*^2^	Secondary atom site location: difference Fourier map
Least-squares matrix: full	Hydrogen site location: inferred from neighbouring sites
*R*[*F*^2^ > 2σ(*F*^2^)] = 0.045	H-atom parameters not refined
*wR*(*F*^2^) = 0.115	*w* = 1/[σ^2^(*F*_o_^2^) + (0.0449*P*)^2^ + 0.4753*P*] where *P* = (*F*_o_^2^ + 2*F*_c_^2^)/3
*S* = 1.08	(Δ/σ)_max_ < 0.001
6509 reflections	Δρ_max_ = 0.57 e Å^−^^3^
506 parameters	Δρ_min_ = −0.27 e Å^−^^3^
0 restraints	Extinction correction: *SHELXL97* (Sheldrick, 2008), Fc^*^=kFc[1+0.001xFc^2^λ^3^/sin(2θ)]^-1/4^
Primary atom site location: structure-invariant direct methods	Extinction coefficient: 0.0038 (7)

### Special details

**Table:**

Geometry. All e.s.d.'s (except the e.s.d. in the dihedral angle between two l.s. planes) are estimated using the full covariance matrix. The cell e.s.d.'s are taken into account individually in the estimation of e.s.d.'s in distances, angles and torsion angles; correlations between e.s.d.'s in cell parameters are only used when they are defined by crystal symmetry. An approximate (isotropic) treatment of cell e.s.d.'s is used for estimating e.s.d.'s involving l.s. planes.
Refinement. Refinement of *F*^2^ against ALL reflections. The weighted *R*-factor *wR* and goodness of fit *S* are based on *F*^2^, conventional *R*-factors *R* are based on *F*, with *F* set to zero for negative *F*^2^. The threshold expression of *F*^2^ > σ(*F*^2^) is used only for calculating *R*-factors(gt) *etc*. and is not relevant to the choice of reflections for refinement. *R*-factors based on *F*^2^ are statistically about twice as large as those based on *F*, and *R*- factors based on ALL data will be even larger.

### Fractional atomic coordinates and isotropic or equivalent isotropic displacement parameters (Å^2^)

**Table:**

	*x*	*y*	*z*	*U*_iso_*/*U*_eq_	
O2	0.35004 (13)	0.62450 (10)	0.15748 (8)	0.0281 (3)	
O52	0.93820 (12)	0.65312 (10)	0.16480 (7)	0.0255 (3)	
N1	0.59643 (16)	0.24086 (12)	0.34929 (10)	0.0286 (4)	
N2	1.08301 (16)	0.23701 (12)	0.35939 (9)	0.0266 (4)	
C1	0.45602 (19)	0.58181 (15)	0.13719 (12)	0.0261 (4)	
C3	0.23645 (19)	0.77012 (15)	0.09996 (12)	0.0283 (4)	
H3	0.1765	0.7605	0.1375	0.034*	
C4	0.2340 (2)	0.84867 (16)	0.03899 (12)	0.0304 (5)	
H4	0.1703	0.8930	0.0347	0.036*	
C5	0.3255 (2)	0.86260 (16)	−0.01633 (12)	0.0296 (4)	
H5	0.3220	0.9166	−0.0565	0.036*	
C6	0.42097 (19)	0.79831 (15)	−0.01288 (12)	0.0283 (4)	
H6	0.4813	0.8083	−0.0502	0.034*	
C7	0.50337 (19)	0.63539 (15)	0.07242 (12)	0.0278 (4)	
H7	0.5739	0.6216	0.0480	0.033*	
C8	0.42488 (19)	0.71795 (15)	0.04790 (12)	0.0253 (4)	
C9	0.33233 (19)	0.70697 (15)	0.10215 (12)	0.0256 (4)	
C11	0.49132 (19)	0.49322 (15)	0.19020 (12)	0.0260 (4)	
C12	0.41322 (19)	0.44974 (15)	0.24970 (12)	0.0272 (4)	
H12	0.3374	0.4774	0.2549	0.033*	
C13	0.44657 (19)	0.36651 (15)	0.30096 (12)	0.0274 (4)	
H13	0.3929	0.3386	0.3402	0.033*	
C14	0.55926 (19)	0.32394 (15)	0.29471 (12)	0.0261 (4)	
C15	0.6363 (2)	0.36514 (16)	0.23456 (12)	0.0301 (5)	
H15	0.7115	0.3367	0.2293	0.036*	
C16	0.60269 (19)	0.44760 (15)	0.18251 (12)	0.0289 (4)	
H16	0.6544	0.4732	0.1419	0.035*	
C21	0.58583 (19)	0.25126 (15)	0.43608 (12)	0.0273 (4)	
C22	0.5392 (3)	0.15753 (18)	0.47949 (15)	0.0480 (6)	
H22	0.5108	0.0839	0.4514	0.058*	
C23	0.5342 (3)	0.1722 (2)	0.56531 (15)	0.0544 (7)	
H23	0.5068	0.1079	0.5946	0.065*	
C24	0.5690 (2)	0.2793 (2)	0.60690 (14)	0.0442 (6)	
H24	0.5653	0.2885	0.6642	0.053*	
C25	0.6091 (2)	0.37278 (19)	0.56317 (13)	0.0389 (5)	
H25	0.6281	0.4464	0.5901	0.047*	
C26	0.6221 (2)	0.36022 (17)	0.47951 (13)	0.0331 (5)	
H26	0.6554	0.4250	0.4520	0.040*	
C41	0.61579 (19)	0.13819 (15)	0.31304 (12)	0.0262 (4)	
C42	0.70935 (19)	0.08269 (16)	0.35718 (13)	0.0306 (5)	
H42	0.7621	0.1139	0.4110	0.037*	
C43	0.7249 (2)	−0.01828 (16)	0.32206 (14)	0.0363 (5)	
H43	0.7872	−0.0550	0.3526	0.044*	
C44	0.6488 (2)	−0.06517 (17)	0.24196 (14)	0.0372 (5)	
H44	0.6589	−0.1335	0.2186	0.045*	
C45	0.5577 (2)	−0.00964 (16)	0.19708 (13)	0.0349 (5)	
H45	0.5072	−0.0400	0.1426	0.042*	
C46	0.54067 (19)	0.09104 (16)	0.23225 (12)	0.0301 (5)	
H46	0.4782	0.1274	0.2014	0.036*	
C51	0.93063 (18)	0.54819 (15)	0.12263 (12)	0.0255 (4)	
C53	0.8815 (2)	0.82966 (17)	0.11816 (13)	0.0349 (5)	
H53	0.9070	0.8674	0.1728	0.042*	
C54	0.8331 (2)	0.8813 (2)	0.04690 (14)	0.0436 (6)	
H54	0.8258	0.9566	0.0534	0.052*	
C55	0.7949 (2)	0.8239 (2)	−0.03435 (14)	0.0430 (6)	
H55	0.7629	0.8617	−0.0809	0.052*	
C56	0.8034 (2)	0.71253 (19)	−0.04757 (13)	0.0359 (5)	
H56	0.7774	0.6749	−0.1022	0.043*	
C57	0.87840 (19)	0.54727 (16)	0.03832 (12)	0.0275 (4)	
H57	0.8623	0.4862	−0.0027	0.033*	
C58	0.85230 (18)	0.65717 (17)	0.02335 (12)	0.0280 (4)	
C59	0.88955 (18)	0.71878 (16)	0.10309 (11)	0.0264 (4)	
C61	0.97725 (18)	0.46656 (15)	0.17885 (11)	0.0233 (4)	
C62	1.05605 (18)	0.50525 (15)	0.26096 (11)	0.0241 (4)	
H62	1.0846	0.5839	0.2775	0.029*	
C63	1.09210 (18)	0.42908 (15)	0.31776 (11)	0.0232 (4)	
H63	1.1456	0.4572	0.3719	0.028*	
C64	1.05031 (18)	0.31045 (15)	0.29604 (12)	0.0236 (4)	
C65	0.97473 (19)	0.27131 (15)	0.21315 (12)	0.0271 (4)	
H65	0.9478	0.1928	0.1962	0.033*	
C66	0.93971 (19)	0.34839 (16)	0.15610 (12)	0.0275 (4)	
H66	0.8898	0.3207	0.1010	0.033*	
C71	1.09740 (19)	0.27741 (14)	0.44662 (12)	0.0251 (4)	
C72	0.99253 (19)	0.31705 (14)	0.47079 (12)	0.0250 (4)	
H72	0.9118	0.3149	0.4306	0.030*	
C73	1.0073 (2)	0.35961 (15)	0.55388 (12)	0.0285 (4)	
H73	0.9368	0.3867	0.5694	0.034*	
C74	1.1263 (2)	0.36231 (16)	0.61449 (12)	0.0313 (5)	
H74	1.1358	0.3907	0.6707	0.038*	
C75	1.2305 (2)	0.32253 (16)	0.59076 (13)	0.0329 (5)	
H75	1.3105	0.3238	0.6313	0.040*	
C76	1.2171 (2)	0.28082 (16)	0.50731 (12)	0.0299 (5)	
H76	1.2884	0.2550	0.4918	0.036*	
C81	1.11014 (18)	0.12962 (15)	0.34196 (12)	0.0249 (4)	
C82	1.0835 (2)	0.04290 (15)	0.39650 (12)	0.0303 (5)	
H82	1.0453	0.0553	0.4430	0.036*	
C83	1.1128 (2)	−0.06090 (17)	0.38260 (13)	0.0385 (5)	
H83	1.0952	−0.1177	0.4200	0.046*	
C84	1.1682 (2)	−0.08133 (17)	0.31368 (14)	0.0395 (5)	
H84	1.1879	−0.1516	0.3043	0.047*	
C85	1.1939 (2)	0.00387 (16)	0.25889 (13)	0.0362 (5)	
H85	1.2304	−0.0096	0.2119	0.043*	
C86	1.16633 (19)	0.10884 (16)	0.27271 (12)	0.0291 (4)	
H86	1.1854	0.1658	0.2356	0.035*	

### Atomic displacement parameters (Å^2^)

**Table:**

	*U*^11^	*U*^22^	*U*^33^	*U*^12^	*U*^13^	*U*^23^
O2	0.0275 (7)	0.0278 (7)	0.0355 (8)	0.0112 (6)	0.0160 (6)	0.0056 (6)
O52	0.0273 (7)	0.0302 (7)	0.0224 (7)	0.0120 (6)	0.0070 (6)	0.0038 (6)
N1	0.0355 (10)	0.0227 (8)	0.0310 (9)	0.0108 (7)	0.0111 (7)	0.0013 (7)
N2	0.0320 (9)	0.0239 (8)	0.0274 (9)	0.0108 (7)	0.0103 (7)	0.0014 (7)
C1	0.0243 (10)	0.0240 (10)	0.0322 (11)	0.0068 (8)	0.0106 (9)	−0.0002 (8)
C3	0.0264 (11)	0.0255 (10)	0.0351 (11)	0.0060 (8)	0.0134 (9)	−0.0018 (9)
C4	0.0279 (11)	0.0266 (10)	0.0369 (12)	0.0093 (8)	0.0053 (9)	0.0016 (9)
C5	0.0305 (11)	0.0260 (10)	0.0316 (11)	0.0054 (8)	0.0071 (9)	0.0041 (8)
C6	0.0276 (11)	0.0297 (10)	0.0280 (11)	0.0056 (8)	0.0092 (9)	0.0017 (8)
C7	0.0257 (11)	0.0268 (10)	0.0335 (11)	0.0079 (8)	0.0116 (9)	0.0005 (8)
C8	0.0225 (10)	0.0226 (9)	0.0299 (11)	0.0035 (8)	0.0071 (8)	−0.0033 (8)
C9	0.0247 (10)	0.0215 (9)	0.0305 (11)	0.0044 (8)	0.0076 (8)	0.0020 (8)
C11	0.0265 (11)	0.0222 (9)	0.0303 (11)	0.0053 (8)	0.0099 (9)	−0.0003 (8)
C12	0.0242 (10)	0.0297 (10)	0.0309 (11)	0.0094 (8)	0.0107 (9)	−0.0008 (9)
C13	0.0280 (11)	0.0272 (10)	0.0280 (11)	0.0061 (8)	0.0100 (9)	0.0013 (8)
C14	0.0271 (11)	0.0204 (9)	0.0309 (11)	0.0055 (8)	0.0078 (9)	−0.0012 (8)
C15	0.0278 (11)	0.0292 (10)	0.0396 (12)	0.0125 (9)	0.0151 (9)	0.0039 (9)
C16	0.0279 (11)	0.0284 (10)	0.0346 (11)	0.0073 (8)	0.0163 (9)	0.0035 (9)
C21	0.0257 (11)	0.0275 (10)	0.0324 (11)	0.0102 (8)	0.0106 (9)	0.0033 (8)
C22	0.0673 (17)	0.0281 (11)	0.0617 (16)	0.0167 (11)	0.0381 (13)	0.0071 (11)
C23	0.0773 (19)	0.0516 (15)	0.0594 (16)	0.0356 (13)	0.0452 (14)	0.0292 (13)
C24	0.0416 (14)	0.0654 (16)	0.0360 (13)	0.0308 (12)	0.0109 (11)	0.0076 (12)
C25	0.0254 (11)	0.0519 (14)	0.0347 (12)	0.0041 (10)	0.0047 (9)	−0.0080 (10)
C26	0.0273 (11)	0.0328 (11)	0.0352 (12)	−0.0004 (9)	0.0084 (9)	−0.0016 (9)
C41	0.0236 (10)	0.0219 (9)	0.0339 (11)	0.0050 (8)	0.0099 (9)	−0.0005 (8)
C42	0.0239 (11)	0.0284 (10)	0.0371 (12)	0.0059 (8)	0.0029 (9)	−0.0025 (9)
C43	0.0268 (11)	0.0299 (11)	0.0551 (14)	0.0120 (9)	0.0098 (10)	0.0010 (10)
C44	0.0369 (12)	0.0268 (10)	0.0506 (14)	0.0078 (9)	0.0179 (11)	−0.0065 (10)
C45	0.0356 (12)	0.0323 (11)	0.0330 (12)	0.0028 (9)	0.0076 (9)	−0.0056 (9)
C46	0.0260 (11)	0.0283 (10)	0.0343 (12)	0.0060 (8)	0.0046 (9)	−0.0001 (9)
C51	0.0211 (10)	0.0282 (10)	0.0287 (11)	0.0049 (8)	0.0108 (8)	0.0016 (8)
C53	0.0373 (12)	0.0411 (12)	0.0347 (12)	0.0215 (10)	0.0122 (10)	0.0083 (10)
C54	0.0504 (14)	0.0519 (14)	0.0431 (14)	0.0330 (12)	0.0173 (11)	0.0182 (11)
C55	0.0401 (13)	0.0634 (15)	0.0363 (13)	0.0285 (12)	0.0118 (10)	0.0217 (11)
C56	0.0261 (11)	0.0575 (14)	0.0265 (11)	0.0134 (10)	0.0080 (9)	0.0078 (10)
C57	0.0225 (10)	0.0336 (11)	0.0258 (11)	0.0047 (8)	0.0079 (8)	−0.0016 (8)
C58	0.0186 (10)	0.0427 (12)	0.0251 (11)	0.0088 (8)	0.0089 (8)	0.0065 (9)
C59	0.0217 (10)	0.0372 (11)	0.0255 (10)	0.0136 (8)	0.0089 (8)	0.0096 (9)
C61	0.0201 (10)	0.0270 (10)	0.0254 (10)	0.0069 (8)	0.0100 (8)	0.0028 (8)
C62	0.0223 (10)	0.0209 (9)	0.0309 (11)	0.0053 (8)	0.0104 (8)	0.0003 (8)
C63	0.0196 (10)	0.0258 (10)	0.0249 (10)	0.0060 (8)	0.0069 (8)	0.0000 (8)
C64	0.0189 (10)	0.0257 (10)	0.0300 (11)	0.0079 (8)	0.0116 (8)	0.0037 (8)
C65	0.0281 (11)	0.0226 (10)	0.0318 (11)	0.0059 (8)	0.0108 (9)	−0.0034 (8)
C66	0.0249 (11)	0.0316 (11)	0.0255 (10)	0.0061 (8)	0.0064 (8)	−0.0013 (9)
C71	0.0295 (11)	0.0201 (9)	0.0276 (10)	0.0067 (8)	0.0102 (9)	0.0021 (8)
C72	0.0262 (10)	0.0223 (9)	0.0281 (10)	0.0067 (8)	0.0085 (8)	0.0040 (8)
C73	0.0307 (11)	0.0236 (10)	0.0340 (11)	0.0077 (8)	0.0128 (9)	0.0016 (8)
C74	0.0345 (12)	0.0306 (11)	0.0285 (11)	0.0066 (9)	0.0096 (9)	−0.0015 (9)
C75	0.0282 (11)	0.0362 (11)	0.0327 (12)	0.0083 (9)	0.0032 (9)	−0.0004 (9)
C76	0.0274 (11)	0.0322 (11)	0.0338 (12)	0.0116 (9)	0.0106 (9)	0.0020 (9)
C81	0.0208 (10)	0.0228 (9)	0.0316 (11)	0.0066 (8)	0.0061 (8)	−0.0016 (8)
C82	0.0314 (11)	0.0273 (10)	0.0323 (11)	0.0078 (8)	0.0067 (9)	0.0030 (9)
C83	0.0486 (14)	0.0260 (11)	0.0401 (13)	0.0138 (10)	0.0023 (11)	0.0044 (9)
C84	0.0436 (13)	0.0273 (11)	0.0486 (14)	0.0173 (10)	0.0037 (11)	−0.0044 (10)
C85	0.0355 (12)	0.0333 (11)	0.0417 (13)	0.0118 (9)	0.0108 (10)	−0.0077 (10)
C86	0.0270 (11)	0.0254 (10)	0.0364 (11)	0.0066 (8)	0.0112 (9)	0.0007 (9)

### Geometric parameters (Å, °)

**Table:**

O2—C9	1.371 (2)	C44—C45	1.375 (3)
O2—C1	1.393 (2)	C44—H44	0.9300
O52—C59	1.375 (2)	C45—C46	1.384 (3)
O52—C51	1.390 (2)	C45—H45	0.9300
N1—C14	1.419 (2)	C46—H46	0.9300
N1—C21	1.420 (2)	C51—C57	1.347 (3)
N1—C41	1.422 (2)	C51—C61	1.453 (2)
N2—C64	1.409 (2)	C53—C59	1.373 (3)
N2—C81	1.417 (2)	C53—C54	1.379 (3)
N2—C71	1.432 (2)	C53—H53	0.9300
C1—C7	1.346 (3)	C54—C55	1.389 (3)
C1—C11	1.452 (3)	C54—H54	0.9300
C3—C9	1.373 (2)	C55—C56	1.376 (3)
C3—C4	1.380 (3)	C55—H55	0.9300
C3—H3	0.9300	C56—C58	1.402 (3)
C4—C5	1.394 (3)	C56—H56	0.9300
C4—H4	0.9300	C57—C58	1.429 (3)
C5—C6	1.379 (3)	C57—H57	0.9300
C5—H5	0.9300	C58—C59	1.390 (3)
C6—C8	1.394 (3)	C61—C66	1.392 (2)
C6—H6	0.9300	C61—C62	1.394 (2)
C7—C8	1.439 (2)	C62—C63	1.373 (2)
C7—H7	0.9300	C62—H62	0.9300
C8—C9	1.391 (3)	C63—C64	1.394 (2)
C11—C12	1.394 (3)	C63—H63	0.9300
C11—C16	1.401 (2)	C64—C65	1.395 (3)
C12—C13	1.377 (3)	C65—C66	1.380 (3)
C12—H12	0.9300	C65—H65	0.9300
C13—C14	1.387 (2)	C66—H66	0.9300
C13—H13	0.9300	C71—C76	1.389 (3)
C14—C15	1.389 (3)	C71—C72	1.387 (2)
C15—C16	1.378 (3)	C72—C73	1.377 (2)
C15—H15	0.9300	C72—H72	0.9300
C16—H16	0.9300	C73—C74	1.384 (3)
C21—C22	1.376 (3)	C73—H73	0.9300
C21—C26	1.391 (3)	C74—C75	1.379 (3)
C22—C23	1.393 (3)	C74—H74	0.9300
C22—H22	0.9300	C75—C76	1.382 (3)
C23—C24	1.361 (3)	C75—H75	0.9300
C23—H23	0.9300	C76—H76	0.9300
C24—C25	1.362 (3)	C81—C86	1.389 (3)
C24—H24	0.9300	C81—C82	1.391 (3)
C25—C26	1.380 (3)	C82—C83	1.375 (3)
C25—H25	0.9300	C82—H82	0.9300
C26—H26	0.9300	C83—C84	1.378 (3)
C41—C46	1.386 (3)	C83—H83	0.9300
C41—C42	1.388 (3)	C84—C85	1.379 (3)
C42—C43	1.380 (3)	C84—H84	0.9300
C42—H42	0.9300	C85—C86	1.380 (3)
C43—C44	1.380 (3)	C85—H85	0.9300
C43—H43	0.9300	C86—H86	0.9300
			
C9—O2—C1	106.04 (13)	C46—C45—H45	119.7
C59—O52—C51	106.23 (14)	C45—C46—C41	120.63 (18)
C14—N1—C21	118.57 (14)	C45—C46—H46	119.7
C14—N1—C41	119.36 (15)	C41—C46—H46	119.7
C21—N1—C41	120.84 (15)	C57—C51—O52	110.47 (16)
C64—N2—C81	123.81 (15)	C57—C51—C61	135.70 (17)
C64—N2—C71	117.42 (14)	O52—C51—C61	113.81 (15)
C81—N2—C71	118.66 (14)	C59—C53—C54	115.53 (19)
C7—C1—O2	110.70 (15)	C59—C53—H53	122.2
C7—C1—C11	135.00 (17)	C54—C53—H53	122.2
O2—C1—C11	114.29 (15)	C53—C54—C55	121.7 (2)
C9—C3—C4	116.33 (17)	C53—C54—H54	119.1
C9—C3—H3	121.8	C55—C54—H54	119.1
C4—C3—H3	121.8	C56—C55—C54	121.55 (19)
C3—C4—C5	121.04 (17)	C56—C55—H55	119.2
C3—C4—H4	119.5	C54—C55—H55	119.2
C5—C4—H4	119.5	C55—C56—C58	118.39 (19)
C6—C5—C4	121.60 (18)	C55—C56—H56	120.8
C6—C5—H5	119.2	C58—C56—H56	120.8
C4—C5—H5	119.2	C51—C57—C58	107.57 (16)
C5—C6—C8	118.39 (17)	C51—C57—H57	126.2
C5—C6—H6	120.8	C58—C57—H57	126.2
C8—C6—H6	120.8	C59—C58—C56	117.65 (18)
C1—C7—C8	107.47 (16)	C59—C58—C57	105.66 (16)
C1—C7—H7	126.3	C56—C58—C57	136.68 (18)
C8—C7—H7	126.3	C53—C59—O52	124.76 (17)
C9—C8—C6	118.27 (16)	C53—C59—C58	125.18 (17)
C9—C8—C7	105.18 (16)	O52—C59—C58	110.05 (15)
C6—C8—C7	136.55 (17)	C66—C61—C62	117.52 (16)
O2—C9—C3	125.02 (16)	C66—C61—C51	122.61 (17)
O2—C9—C8	110.61 (15)	C62—C61—C51	119.76 (16)
C3—C9—C8	124.36 (17)	C63—C62—C61	121.01 (16)
C12—C11—C16	117.99 (17)	C63—C62—H62	119.5
C12—C11—C1	120.54 (16)	C61—C62—H62	119.5
C16—C11—C1	121.46 (17)	C62—C63—C64	121.49 (17)
C13—C12—C11	121.06 (17)	C62—C63—H63	119.3
C13—C12—H12	119.5	C64—C63—H63	119.3
C11—C12—H12	119.5	C63—C64—C65	117.80 (16)
C12—C13—C14	120.69 (17)	C63—C64—N2	118.39 (16)
C12—C13—H13	119.7	C65—C64—N2	123.79 (16)
C14—C13—H13	119.7	C66—C65—C64	120.41 (17)
C13—C14—C15	118.72 (17)	C66—C65—H65	119.8
C13—C14—N1	120.79 (17)	C64—C65—H65	119.8
C15—C14—N1	120.48 (16)	C65—C66—C61	121.71 (17)
C16—C15—C14	120.88 (17)	C65—C66—H66	119.1
C16—C15—H15	119.6	C61—C66—H66	119.1
C14—C15—H15	119.6	C76—C71—C72	119.18 (17)
C15—C16—C11	120.61 (17)	C76—C71—N2	120.89 (16)
C15—C16—H16	119.7	C72—C71—N2	119.90 (16)
C11—C16—H16	119.7	C73—C72—C71	120.38 (17)
C22—C21—C26	117.98 (18)	C73—C72—H72	119.8
C22—C21—N1	122.68 (17)	C71—C72—H72	119.8
C26—C21—N1	119.34 (17)	C72—C73—C74	120.42 (18)
C21—C22—C23	120.4 (2)	C72—C73—H73	119.8
C21—C22—H22	119.8	C74—C73—H73	119.8
C23—C22—H22	119.8	C75—C74—C73	119.36 (18)
C24—C23—C22	121.0 (2)	C75—C74—H74	120.3
C24—C23—H23	119.5	C73—C74—H74	120.3
C22—C23—H23	119.5	C74—C75—C76	120.56 (18)
C23—C24—C25	118.8 (2)	C74—C75—H75	119.7
C23—C24—H24	120.6	C76—C75—H75	119.7
C25—C24—H24	120.6	C75—C76—C71	120.09 (17)
C24—C25—C26	121.2 (2)	C75—C76—H76	120.0
C24—C25—H25	119.4	C71—C76—H76	120.0
C26—C25—H25	119.4	C86—C81—C82	118.53 (16)
C25—C26—C21	120.42 (19)	C86—C81—N2	121.87 (16)
C25—C26—H26	119.8	C82—C81—N2	119.58 (16)
C21—C26—H26	119.8	C83—C82—C81	120.81 (19)
C46—C41—C42	118.41 (17)	C83—C82—H82	119.6
C46—C41—N1	120.36 (16)	C81—C82—H82	119.6
C42—C41—N1	121.24 (17)	C82—C83—C84	120.50 (19)
C43—C42—C41	120.65 (18)	C82—C83—H83	119.8
C43—C42—H42	119.7	C84—C83—H83	119.8
C41—C42—H42	119.7	C85—C84—C83	119.06 (18)
C44—C43—C42	120.54 (19)	C85—C84—H84	120.5
C44—C43—H43	119.7	C83—C84—H84	120.5
C42—C43—H43	119.7	C84—C85—C86	120.97 (19)
C45—C44—C43	119.21 (18)	C84—C85—H85	119.5
C45—C44—H44	120.4	C86—C85—H85	119.5
C43—C44—H44	120.4	C85—C86—C81	120.13 (18)
C44—C45—C46	120.54 (19)	C85—C86—H86	119.9
C44—C45—H45	119.7	C81—C86—H86	119.9

### Hydrogen-bond geometry (Å, °)

**Table:**

Cg is the centroid of the C61–C66 ring.

**Table:**

*D*—H···*A*	*D*—H	H···*A*	*D*···*A*	*D*—H···*A*
C15—H15···Cg	0.93	2.97	3.787 (2)	147
